# Overview of Glutamatergic Dysregulation in Central Pathologies

**DOI:** 10.3390/biom5043112

**Published:** 2015-11-11

**Authors:** Tanya Miladinovic, Mina G. Nashed, Gurmit Singh

**Affiliations:** Department of Pathology and Molecular Medicine, McMaster University, 1280 Main Street West, Hamilton, ON L8S 4L8, Canada; E-Mails: raaphot@mcmaster.ca (T.M.); nashedm@mcmaster.ca (M.G.N.)

**Keywords:** glutamate, glutamate dysregulation, psychiatric disorder, neurodegenerative disease, excitotoxicity

## Abstract

As the major excitatory neurotransmitter in the mammalian central nervous system, glutamate plays a key role in many central pathologies, including gliomas, psychiatric, neurodevelopmental, and neurodegenerative disorders. Post-mortem and serological studies have implicated glutamatergic dysregulation in these pathologies, and pharmacological modulation of glutamate receptors and transporters has provided further validation for the involvement of glutamate. Furthermore, efforts from genetic, *in vitro*, and animal studies are actively elucidating the specific glutamatergic mechanisms that contribute to the aetiology of central pathologies. However, details regarding specific mechanisms remain sparse and progress in effectively modulating glutamate to alleviate symptoms or inhibit disease states has been relatively slow. In this report, we review what is currently known about glutamate signalling in central pathologies. We also discuss glutamate’s mediating role in comorbidities, specifically cancer-induced bone pain and depression.

## 1. Introduction

Glutamate is the major excitatory neurotransmitter in the central nervous system (CNS). Central and peripheral glutamate signalling includes multiple transporters, as well as ionotropic and metabotropic receptors [1,2,3]. In addition to its critical role in the CNS, glutamate also contributes to autocrine and paracrine signalling in peripheral tissues, such as bone, testis, pancreas, and the adrenal, pituitary and pineal glands [4]. Glutamate also plays an import role in peripherally-mediated pain signalling to the CNS [5]. Considering glutamate’s widespread role in important central and peripheral processes, it is unsurprising that glutamate release, uptake, metabolism, and signalling are tightly regulated processes. Disturbances in these processes are often aetiologically associated with central pathologies [6].

Glutamate dysregulation has been well characterized in certain psychiatric, neurodevelopmental, and neurodegenerative disorders, such as schizophrenia, fragile X syndrome, and epilepsy [7,8]. The emerging role of glutamate signalling in other disorders, such as depression and anxiety, has also garnered recent attention and led to novel hypotheses of glutamate dysregulation [7]. However, psychiatric and neurodegenerative disorders are complex disease states and likely arise from multiple related processes. Therefore, progress in understanding the impact of glutamate dysregulation on central pathologies is predictably slow and, at times, tenuous. Elucidating mechanisms is equally complicated in comorbid conditions where glutamate may play a key mediating role. Gliomas are known to release large amounts of glutamate through the cystine/glutamate antiporter system x_c_^−^ [9]. Considering the role of glutamate dysregulation in other central pathologies, it is plausible that glutamate is an important mediating molecule in comorbidities, such as cancer-induced bone pain and depression [10,11]. Here, we provide an overview of what is currently known regarding glutamate modulation in central pathologies.

## 2. Glutamate Receptors and Transporters

Glutamate is an important signalling molecule and a major excitatory neurotransmitter in the mammalian CNS [12]. Its receptors are localized on both neuronal and non-neuronal cells and regulate a broad range of processes [13]. Under normal physiological conditions, it is released as a neurotransmitter into the synaptic cleft and initiates the propagation of action potentials. Glutamate is largely found intracellularly, with relatively little (up to one million-fold less) found in the extracellular environment. This establishes the steep concentration gradient required for rapid synaptic transmission [12,14,15].

Astrocytes readily convert glutamine to glutamate via the glutamine synthetase pathway. Glutamine is then released from astrocytes, taken up into presynaptic terminals, metabolised into glutamate by the mitochondrial enzyme glutaminase, and packaged into synaptic vesicles [16].

Considerable metabolic commitment is devoted to regulating glutamate metabolism, uptake, and release [17]. Glutamate levels are tightly regulated by sodium-dependent glutamate transporters, which remove excess glutamate molecules from the extracellular fluid, and are predominantly present on surrounding astrocytes at the synapse [18,19,20,21]. Glutamate transporters maintain low concentrations of synaptic and extrasynaptic glutamate by using the established electrochemical gradients as the driving force for uptake to protect against excitotoxicity. At elevated extracellular concentrations, glutamate becomes neurotoxic and capable of degenerating target neurons. Under pathological conditions, extracellular glutamate exceeds normal levels and ionotropic glutamate receptors are overactive, triggering excitotoxicity and cell death in surrounding postsynaptic neurons [22,23].

### 2.1. Glutamate Receptors

Through molecular cloning, several types of glutamate receptors (GluRs) have been identified. Glutamate-gated ion channels comprise the ionotropic glutamate receptors (iGluRs). These ligand-gated ion channels include *N*-methyl-D-aspartate receptors (NMDARs), α-amino-3-hydroxy-5-methyl-4- isoxazole propionic acid receptors (AMPARs), and kainate receptors, named after the synthetic agonists that activate them. Like other ligand-gated channel receptors, iGLuRs are formed from the association of several protein subunits that combine in various ways to produce a large number of receptor isoforms.

In addition to the iGluRs, metabotropic glutamate receptors (mGluRs) indirectly modulate postsynaptic ion channels, consist of G-protein coupled receptors (mGluR1-8), which are further subdivided according to their activation by either (±)1-amino-cyclopentane-trans-1,3-dicarboxylic acid (trans-ACPD) or L(+)-2-amino-4-phosphonobutyric acid (L-AP4), and cause slower synaptic responses that can either increase or decrease postsynaptic potentials [24,25,26,27].

### 2.2. Glutamate Transporters

To date, five plasma membrane glutamate transporter subtypes have been cloned: the excitatory amino acid transporters (EAATs) GLAST/EAAT1, GLT1/EAAT2, EAAC1/EAAT3, EAAT4, and EAAT5 [28,29,30,31,32]. GLAST/EAAT1 is found primarily on astrocytes and oligodendrocytes and is the major glutamate transporter in the forebrain [33], GLT1/EAAT2 is found on astrocytes throughout the CNS [33], EAAC1/EAAT3 is found primarily on neurons in the hippocampus, basal ganglia, and cerebellum [33,34], EAAT4 is found on Purkinje cells [33,34], and EAAT5 on photoreceptors and bipolar cells [32].

The cystine/glutamate antiporter system x_c_^−^ is predominantly expressed on glial cells and is a critical regulator of non-synaptic extracellular glutamate levels [35]. System x_c_^−^, a transporter that catalyses a 1:1 stoichiometric release of glutamate in exchange for cystine uptake, is composed of two subunits: a catalytic subunit light-chain xCT and a heavy-chain glycoprotein 4F2hc [36]. Imported cystine is then reduced to cysteine, which is used in the intracellular production of the antioxidant glutathione (GSH) [37]. GSH plays an important role in neutralizing reactive oxygen species (ROS), which are a byproduct of oxidative metabolism in the CNS [38].

## 3. Glutamate Dysregulation in Psychiatric Disorders

### 3.1. Mood and Anxiety Disorders

Mood and anxiety disorders account for most psychiatric diagnoses, and their disease burden outweighs cardiovascular disease, diabetes, and lung cancer [39]. Major depressive disorder (MDD) is the most common mood disorder, with a lifetime prevalence of ~8%–12% [40]. In 2000, Berman and colleagues were first to report on the clinical antidepressant effects of ketamine, an antagonist to NMDARs [41]. More recently, single doses of ketamine have been robustly demonstrated to alleviate depressive symptoms for up to one week, even in treatment-resistant patients [42,43,44]. The involvement of glutamatergic dysregulation in MDD has been further corroborated by the observation that glutamine is elevated in the cerebrospinal fluid (CSF) [45], and glutamate is elevated in the serum/plasma of depressed patients [46]. At the brain level, it is likely that changes in glutamatergic signalling are region-specific. For example, results from magnetic resonance spectroscopy (MRS) suggest decreased levels of glutamate in the prefrontal cortex (PFC) and anterior cingulate cortex (ACC) of patients with MDD [47,48]. In addition to clinical observations, post-mortem hippocampal samples from depressed subjects were shown to contain decreased expression of genes that encode subunits of AMPARs [49]. Furthermore, animal studies have shown that induction of physiological stress causes release and accumulation of glutamate in the hippocampus [50,51], an effect which is attenuated by adrenalectomy [52]. Deficiencies in glial cell density and neuronal size have also been reported in the PFC, amygdala, and ACC of depressed subjects [53,54,55]. Glial cells help regulate extracellular glutamate concentrations by taking up and storing glutamate as glutamine. Therefore, glial cell deficiency likely contributes to glutamate accumulation and subsequent excitotoxicity at synapses. Although the precise aetiological role of glutamatergic dysregulation in depression remains unknown, pre-clinical studies have provided some answers. In rats, administration of ketamine caused rapid activation of the mammalian target of rapamycin (mTOR) pathway, which is involved in synaptogenesis, and blocking mTOR signalling effectively blocked ketamine-induced synaptogenesis [56]. It is likely that antagonism of NMDARs causes fast excitation through AMPARs, leading to downstream release of brain-derived neurotrophic factor (BDNF) and activation of kinases, which in turn stimulate mTOR (reviewed by Duman and Voleti [57]).

Bipolar disorder (BPD) is another major mood disorder, which is characterized by alternating periods of depressed mood and periods of mania or hypomania. BPD is classically treated with several types of psychoactive drugs [58]. Most notably, treatment of BPD with the mood stabilizer lithium over the past 50 years has proven to be the most efficacious long-term management option currently available [59,60]. Anticonvulsants, such as carbamazepine, are also often used as mood stabilizers independently or in combination with lithium [58]. More recently, antipsychotics, particularly olanzapine, quetiapine, risperidone, and haloperidol, have demonstrated superior efficacy compared to mood stabilizers in the acute treatment of manic episodes [61]. Antidepressants have also been used in BPD, although their use has been controversial due to scarce evidence of their efficacy in bipolar depression [62,63,64]. Although some anticonvulsants and antidepressants have been shown to modulate glutamate transmission [65], these effects have been incidental rather than deliberate targeting. Until recently, few studies had investigated glutamate transmission in BPD, despite some early evidence showing NMDAR dysfunction in the hippocampus of patients with BPD [66]. However, recent insight into the importance of glutamate transmission in unipolar depression has generated new interest in the involvement of similar mechanisms in bipolar depression. Randomized controlled trials investigating ketamine’s efficacy in treatment-resistant bipolar depression have yielded similar results to those seen in unipolar depression studies, with rapid and robust antidepressant effects achieved after a single-dose [67,68]. Emerging evidence from MRS studies has provided further insight into glutamatergic dysregulation in BPD, showing decreased glutamate in the hippocampus and increased glutamate in the ACC, frontal regions, and whole brain [65,69,70]. Elevated serum glutamate has also been observed in BPD, although this could be attributed to the use of anticonvulsants [71]. In addition to the altered levels of glutamate in the brain, post-mortem hippocampal samples from subjects with BPD reveal decreased transcript expression of the NR1 and NR2A NMDAR subunits [72,73]. Polymorphisms in the genes encoding NR1, NR2A, and NR2B subunits have also been linked to increased BPD susceptibility [72].

Anxiety disorders are often comorbid with depression [74]. Selective serotonin reuptake inhibitors (SSRIs), the most commonly used class of antidepressants, are the first line of treatment for anxiety disorders [75]. It is therefore reasonable to expect overlap between aetiological mechanisms of depression and anxiety. Although the role of γ-aminobutyric acid (GABA), the major inhibitory neurotransmitter in the CNS, has been extensively studied in anxiety disorders, glutamatergic transmission has received less attention. A recent MRS study in adolescents with generalized anxiety disorder (GAD) found that the ratio of glutamate to creatine (Glu/Cr) in the ACC correlated with severity of anxiety scores [76]. In preclinical murine models, ketamine has been shown to alleviate anxiety in a model of posttraumatic stress disorder (PTSD) [77], although chronic NMDAR antagonism with ketamine and memantine in previously healthy animals paradoxically induces an anxiety-like phenotype [78,79]. MRS studies have also investigated glutamate levels in patients with obsessive-compulsive disorder (OCD). Cumulatively, these studies suggest that glutamate is decreased in the ACC and orbitofrontal cortex (OFC) of patients with OCD, although results from other brain regions, such as the head of the caudate (HOC), were mixed [80,81]. Despite region-specific decreases in glutamate, total brain levels of glutamate are elevated in the CSF of OCD patients [82,83]. Similar to other anxiety and mood disorders, ketamine and other NMDAR antagonists have shown beneficial effects in alleviating OCD symptoms [84]. Riluzole, which stimulates glutamate uptake by astrocytes and inhibits presynaptic glutamate release, has also demonstrated efficacy in decreasing OCD symptoms [85]. Genetic studies further support the role of glutamatergic dysregulation in OCD, with several polymorphisms of glutamate receptor subunits and transporters being associated with increased OCD susceptibility (reviewed by Kariuki-Nyuthe *et al.* [85]).

### 3.2. Schizophrenia

Although less common than mood and anxiety disorders, schizophrenia (SZ) accounts for alarmingly high human and financial costs, with patients experiencing mortality 12–15 years sooner than the general community [86]. SZ is characterized by a set of positive and negative symptoms. Positive psychotic symptoms include delusions and hallucinations, whereas negative symptoms represent deficits, such as social withdrawal and lack of motivation [87]. Mood symptoms might also occur in conjunction with psychotic symptoms, in which case a diagnosis of schizoaffective disorder may be made [88].

At the neurochemical level, SZ research has largely focused on the role of dopamine and glutamate transmission. Early evidence showing reduced levels of glutamate in the CSF of SZ patients implicated glutamatergic dysregulation in the aetiology of the disorder [89]. This hypothesis was strengthened by observations that NMDAR antagonists, such as ketamine and phencyclidine (PCP), induce psychotomimetic symptoms in both human subjects and animal models, suggesting that NMDARs are hypofunctional in SZ [90,91,92,93]. Despite early evidence of reduced CSF glutamate in SZ, more recent MRS studies have found increased glutamate levels in the PFC, basal ganglia, and hippocampus [94], which may explain volumetric reductions of these brain regions through glutamatergic excitotoxicity [95]. To explain the paradoxical observations of NMDAR hypofunction and glutamatergic excitotoxicity in SZ, animal studies have suggested that NMDAR hypofunction at GABA-ergic inhibitory interneurons is sufficient to induce SZ symptoms [96]. Parvalbumin-expressing GABA-ergic interneurons inhibit cortical pyramidal neurons. Therefore, NMDAR hypofunction would cause disinhibition of pyramidal neurons, leading to increased glutamate release and the observed excitotoxicity [95,97]. Post-mortem studies of brain tissue from SZ patients have examined glutamatergic neuron morphology, as well as glutamatergic receptor and transporter mRNA expression in relevant brain regions such as the frontal cortex, temporal lobe, and hippocampus (reviewed by Hu *et al.* [98]). Studies examining neuronal morphology have generally revealed decreased dendritic length, number, and spine density, as well as decreased synaptophysin protein expression. However, studies examining transcriptional control of glutamate receptors and transporters have yielded largely mixed results, with few studies following up mRNA results with protein expression analysis [98]. The genetic impact on the aetiology of SZ has been investigated, with several candidate genes proposed to confer increased susceptibility [99]. The increasing focus on the role of glutamate in SZ has led to several investigations into polymorphisms associated with glutamatergic signalling in particular. Converging results from these investigations suggest that polymorphisms of *GRM7*, which encodes mGluR7, are associated with SZ susceptibility [100,101].

### 3.3. Autism Spectrum Disorder

Autism spectrum disorder (ASD) is a neurodevelopmental disorder that affects nearly 22 million people globally [102]. Fragile X syndrome (FXS) is a heritable syndrome characterized by intellectual disability, and is the most common genetic cause of ASD [103]. The genetic correlate of FXS is the silencing of the *FMR1* gene, which encodes the fragile X mental retardation protein (FMRP). In recent years, investigations into the downstream effects of impaired FMRP expression have revealed deficits in mGluR-dependent long-term depression (LTD) and long-term potentiation (LTP), which are critical neuronal processes in learning [8]. Direct evidence from ASD animal studies has corroborated results from FXS studies, showing that mGluR5, in particular, is relevant to the pathogenesis of ASD (reviewed by Zantomio *et al.* [104]). Other polymorphisms for glutamate receptors have been associated with ASD, such as polymorphisms in *GluR6* [105]. In addition to genetic studies, increased levels of serum glutamate have been reported in ASD patients [106]. MRS studies have provided further validation for glutamatergic involvement in ASD, with evidence for increased glutamate in the ACC and decreased glutamate in the frontal and occipital lobes, although results were not robust across studies [81].

### 3.4. Attention-Deficit/Hyperactivity Disorder

Attention-deficit/hyperactivity disorder (ADHD) is another common neurodevelopmental disorder that affects approximately 39 million people globally [102]. Genome-wide association studies have revealed several glutamate receptor/transporter polymorphisms that are associated with ADHD, including *GRM7*, *GRIN2A*, *GRIN2B*, *GRID2*, and *EAAT1* [107]. In addition, animal studies targeting *GRM5* using pharmacological inhibition or knockout models report locomotor hyperactivity and other impairments, such as deficits in spatial learning (reviewed by Lesch *et al.* [107]). MRS studies on patients with ADHD have reported increased glutamate levels in PFC, ACC, and striatum [81].

## 4. Glutamate Dysregulation in Neurodegenerative Disease

Relatively little is known about the molecular mechanisms that mediate the expression of glutamate transporters and cause aberrant signalling in neurodegenerative disease. Across various acute and chronic neurodegenerative conditions, studies suggest that glutamate transport dysregulation is implicated and largely attributable to decreased transporter protein level. Protein downregulation is found in chronic neurodegenerative diseases such as Alzheimer’s disease (AD) [108,109], Huntington’s disease (HD) [110,111], Parkinson’s disease (PD) [112,113], amyotrophic lateral sclerosis (ALS) [114,115,116], as well as acute conditions including ischemia/hypoxia [117,118,119,120,121,122,123,124]. However, the mechanisms underlying such protein loss are poorly understood, and the downregulation of transporter protein is not always consistent with that of its corresponding mRNA expression. For example, amyloid precursor protein (APP) plays a central role in Alzheimer’s disease. Transgenic mice expressing a mutant form of this protein exhibit decreased glutamate uptake activity and transporter protein levels, but normal transporter mRNA levels [109]. Interestingly, a post-mortem analysis of Alzheimer’s patients’ prefrontal cortices revealed the same results [108].

### 4.1. Epilepsy

Epileptic seizures have many possible causes. However, irrespective of the primary pathology, glutamate seems to be implicated in generating and propagating epileptic discharges. Seizures can be provoked by various glutamatergic molecular mechanisms in epileptic and non-epileptic patients and animals [125]. Compelling neurophysiological, pharmacological, biochemical, and anatomical evidence implicates iGluR- and mGluR-mediated mechanisms in epilepsy.

Inhibiting glutamate transporter synthesis produces elevated extracellular glutamate levels that may lead to excitotoxicity. The use of glutamate transporter knockout mice has provided direct evidence for the involvement of glutamate in epilepsy. Homozygous EAAT2 knockout mice display neuronal loss, physiological changes in the hippocampus, and experience lethal seizures [126]. Likewise, antisense knockdown of EAAT3 induces epileptic seizures and neurotoxicity [123], and EAAT1 knockout mice show more severe seizures than their wild type counterparts [127].

The role of glutamate in epilepsy may also be an indirect result of GABA metabolism, as glutamate is the precursor for GABA synthesis. The knockdown of EAAT3 is associated with a 50% loss of hippocampal GABA, and reduced EAAT3 expression by antisense treatment leads to behavioural abnormalities and an impairment in newly synthesized GABA from extracellular glutamate [128]. In an effort to investigate the relationship between epilepsy and glutamate, animal models of epilepsy and human tissues have been utilized to quantify EAAT3 and other transporter expression, with variable results that are seemingly dependant on both the paradigm and brain region under study. Cumulatively, epilepsy models revealed a downregulation of the neuronal transporter EAAT3 in hippocampal subregions [129], and increases in EAAT3 protein in the Golgi complex and plasma membranes [130]. Conversely, amygdala-kindling induced increased EAAT3 in the piriform cortex and hippocampus [131], and a model of temporal lobe epilepsy in rats demonstrated a threefold increase in the expression of EAAT3 in single dissociated dentate granule cells in epileptic rats when compared to controls [132].

In human patients with epilepsy, hippocampal microdialysis revealed elevated levels of glutamate following epileptic activity. This represented an important milestone in ictal research, as it suggested that alterations in glutamate metabolism might play a role in epileptic activity, either from increased release, decreased transport, or as a combination [133].

In patients undergoing temporal lobe lobectomy for temporal lobe epilepsy, no significant changes in EAAT1 and EAAT2 mRNA and protein levels were observed in the hippocampus or medial temporal cortex [134]. Likewise, astroglial EAAT2 levels were unchanged by temporal lobe epilepsy but EAAT3 mRNA and protein levels increased in these tissues. Anatomical analysis of human hippocampi from temporal lobe epilepsy patients also showed region-specific changes in EAAT1–3 [132].

These collective changes in glutamate transporter expression may exist as a compensatory mechanism for excess synaptic glutamate. The role of glutamate in ictal activity is well established and the study of glutamate transport is of particular importance given the epileptic properties of EAAT2 knockout mice. Epileptic research allows for a unique and potentially more reliable approach than other neurological diseases, as it has the potential of obtaining fresh human tissue from temporal lobectomy, rather than relying on inferences from animal studies or post-mortem human tissue.

### 4.2. Alzheimer’s Disease

Studies of AD in animal models and human tissue suggest that aberrant functioning of the glutamate uptake system is involved in the disease [108,135]. APP plays a central role in AD and protects against excitotoxicity by regulating the function of glial glutamate transporters [136]. Transgenic mice expressing a mutant form of the human APP showed decreased expression of EAAT1 and EAAT2, but respective mRNA levels remained unaffected, suggesting that mutant forms of APP disrupt astroglial transport of excitatory amino acids at the posttranscriptional level and introduce vulnerability to glutamate toxicity [109].

An alternative hypothesis for the role of glutamate transporters in AD neuronal dysfunction is supported by an *in vitro* model of AD. Astrocyte cultures treated with amyloid-beta peptide (Aβ) showed increased extracellular glutamate clearance and decreased glutamatergic synaptic function in cultured neurons, suggesting redistribution in EAAT1 to the astrocyte membrane [137].

Another study focused on activated microglia, rather than astrocytes. The authors speculate that the activation of microglia by Aβ increases extracellular glutamate through reversal of glutamate transport, and that this mechanism may contribute to the pathological neuronal dysfunction and death in AD [138]. Furthermore, aberrant expression of EAAT1, a glutamate transporter typically expressed exclusively on astroglia, was seen on neurons of AD patients and was closely associated with the distribution of tau-positive cells and neurofibrillary changes [139]. Similarly, EAAT2-immunoreactive neurons with tau deposits characteristic of neuronal pathology are present in brain tissue of AD patients [140]. This altered glutamate expression may be a compensatory reaction to increased extracellular glutamate, and may be indicative of a secondary phenomenon related to impaired cells, or it may play a central role in the propagation of AD.

In a post-mortem analysis of AD patients’ frontal cortices, EAAT2-immunoreactivity, but not mRNA expression, was significantly decreased when compared to controls. Further, an inverse correlation was found between EAAT2 and APP isoform mRNA levels, suggesting a relationship between aberrant glutamate transport and APP processing of amyloidogenic fragments [108]. Decreased functional glutamate transporter activity is also associated with increased excitotoxicity and neurodegeneration [135].

The aforementioned studies focus on various aspects of glutamate dysregulation in AD, and cumulatively implicate multiple pathways of glutamate transporter trafficking and physiology that may be involved in AD, including modulation of transporter function, glutamate transporter reversal, and posttranscriptional modification. The pathophysiology of AD is complex and has yet to be fully elucidated, but glutamate dysregulation and the resulting excitotoxicity at NMDA receptors appear to be contributing factors. Memantine, a non-competitive NMDA receptor antagonist, blocks the NMDA receptor channel, thereby reducing the background noise present in pathological glutamate signalling and therefore facilitates normal physiological function. In accordance with this, memantine and other partial NMDA antagonists have been used alone or in combination with cholinesterase inhibitors to modulate the pathological affects of glutamate in AD patients [141].

### 4.3. Huntington’s Disease

Like many of the pathologies discussed in this review, research implicating glutamate transport in HD is limited. In early studies, no systemic defects were observed in patients with HD [142], however, in a small post-mortem study using three brains of human AD patients, a reduction of EAAT2 mRNA was found in the neostriatum and the degree of reduction correlated with disease progression. In accordance with other neurodegenerative diseases, an increase was seen in the number of astrocytes with EAAT2 transcripts [143].

In a transgenic mouse model of HD, movement disorder with associated central pathology resulted from the polyglutamine repeat. Following the onset of movement disorder, a decrease in striatal and cortical EAAT2 mRNA and associated glutamate decrease was observed, implicating EAAT2 in the cascade of neuronal death [110]. A second model of HD using mutant R6/2 mice showed an age-dependent downregulation of EAAT2 mRNA and protein and a progressive reduction in transporter function [111].

### 4.4. Parkinson’s Disease

Recent studies suggest the involvement of malfunctioning glutamate transporters in the progression of PD, but evidence for a direct role of glutamate dysregulation is less established than in other models. One possible contributor to the pathogenesis of PD is impaired glutamate transport and subsequent increased glutamate to basal ganglia output nuclei. In cultured astrocytes, 1-Methyl-4-phenyl-1,2,3,6-tetrahydropyridine (MPTP), a Parkinsonian neurotoxin known to target dompaminergic neurons, reversibly inhibits astroglial uptake of glutamate [144]. Furthermore, studies of the motor circuitry implicated in PD have provided important clues about the role of glutamate transporter regulation. In an effort to disrupt striatal glutamatergic innervation, Ginsberg and colleagues employed unilateral aspiration of the cerebral cortex-corpus callosum pathway, and demonstrated a reduction in EAAT2 protein expression and EAAT1 activity by 50 and 40%, respectively [112]. Similarly, cortical lesions cause the downregulation of these transporters and an associated reduction in overall glutamate transport within the striatum [113].

In contrast, bilateral cortical thermalcoagulation increased striatal EAAT2 mRNA and protein expression but decreased total glutamate transport [145]. Furthermore, the NMDAR agonist (*S*)-(β-*N*-methylamino) alanine (BMA-A) induced motor neuron dysfunction and Parkinsonian features in monkeys [146]. Cumulatively, these models offer insight to the involvement of glutamate transporter expression in PD, and the possibility of manipulating expression in the nigrostriatal system as a potential pharmacological intervention.

### 4.5. Amyotrophic Lateral Sclerosis (ALS)

The identification of mutations in the SOD1 gene has led to a significant breakthrough in the understanding of ALS aetiological mechanisms [147,148]. Transgenic mice expressing a mutant form of SOD1 develop paralysis and spinal neuron degeneration characteristic of ALS [114,149]. SOD1 transgenic mice [114] and rats [115] also show a reduction in the glutamate transporter EAAT2. In human patients with ALS, glutamate levels in CSF are elevated, likely due to the loss of the EAAT2 protein in motor cortex and spinal cord [116,150,151]. While mounting evidence suggests that glutamate dysregulation is not the primary source of ALS, glutamate transporters are seemingly implicated in the propagation of motor neuron loss.

### 4.6. Stroke and Ischemia

Multiple animal models of ischemia have been applied to study glutamate transporter expression, and have yielded variable outcomes, making it difficult to interpret results. In ischemia, glutamate transporters reverse operation and subsequently give rise to extracellular glutamate, excitotoxicity, and ischemic insult [152]. Hypoxia-ischemia initiates the downregulation of neuronal EAAT3 and astroglial EAAT2 [120]. Following ischemia, EAAT2 mRNA and protein levels are modestly decreased in rat hippocampus [153], but neuronal and oligodendroglial EAAT3 levels are transiently increased. EAAT1 and EAAT2 levels in CA1 and layer V pyramidal neurons of cerebral cortex remain unaffected [154].

Glutamate transporter expression in human hypoxic-ischemic injury has not been extensively studied, but a preliminary study in human neonates showed reduced EAAT1 and EAAT4 glutamate transporter expression in the cerebellum. Given that the cerebellum is particularly dense with EAAT1, and that EAAT4 expression is specific to Purkinje cells, the authors suggest that this reduction may account for the selective vulnerability of Purkinje cells to hypoxia-ischemic injury [119]. A post-mortem study of human autopsy cases showed weak EAAT2 expression in the striatum, cortex, and hippocampus, but the variability in post-mortem delay was significantly different between samples, making it difficult to draw reliable conclusions [155].

In the late 1980s, the high-affinity non-competitive NMDA antagonist dizocilpine (MK-801) was shown to significantly reduce histological lesions in a rodent model of focal cerebral ischemia, which triggered widespread development of NMDA antagonists for the treatment of stroke. Clinically, a myriad of glutamate modulatory therapies are now used to treat stroke, with variable efficacy and associated side effects. Given glutamate’s critical role in normal physiological functions, it is not surprising that total blockade of glutamate signalling has led to serious adverse effects. Thus, partial NMDA receptor antagonists, including memantine [156] and the disulfiram metabolite DETC-MeSO [157], have been developed to maintain basal glutamate transmission while mitigating adverse side effects.

## 5. Glutamate Dysregulation in Gliomas

Gliomas are the most prevalent type of brain tumour, representing approximately one third of all primary CNS tumours and over 80% of malignant CNS tumours [158]. Gliomas arise from the glial cells in the CNS, most commonly astrocytes, and are typically high-grade malignant tumours [159,160].

Under normal physiological conditions, glial cells play an important role in regulating glutamate transmission. Excess glutamate uptake by nearby astrocytes represents the predominant mechanism of glutamate clearance and degradation from synaptic clefts. Glutamate is taken up via EAAT1 and EAAT2, and is subsequently converted to glutamine by glutamine synthetase [161]. Glutamine can then be released from astrocytes to be taken up by neurons, where it is converted back into glutamate by glutaminase and packaged into synaptic vesicles. Astrocytes also express the cystine/glutamate antiporter system x_c_^−^, which plays an important role in the production of the antioxidant GSH [162,163].

Glutamatergic dysregulation in the growth, progression, and metastasis of glioma has been well established through *in vitro*, animal, and clinical evidence (reviewed by de Groot and Sontheimer [161], Robert and Sontheimer [164], Sontheimer [165,166], and Willard and Koochekpour [167]). Glioma cells have been demonstrated to release excitotoxic levels of glutamate *in vitro* [9], which is now accepted to be primarily mediated through system x_c_^−^ transport [168,169,170,171]. More recently, other types of cancer cells have also been demonstrated to release excess amounts of glutamate through system x_c_^−^, most notably melanoma, breast, and prostate carcinomas [172]. In glioma cells, dysregulation of glutamate confers an adaptive advantage that is mediated in several ways. Increased uptake of cystine, and consequently GSH production, enables rapid detoxification of ROS [164]. This provides protection for the cells against the oxidative stress produced by their own rapid proliferation, therefore enhancing growth and survival. The resulting efflux of glutamate accumulates in the extracellular environment due to EAAT deficiency in glioma cells, possibly owing to mGluR5 overexpression [164,167]. Furthermore, excitotoxicity through over-activation of NMDARs has been shown to cause nearby neuronal cell death, creating physical space in which the tumour is able to expand [166,173]. Glutamate secreted by glioma cells has also been shown to act as an autocrine/paracrine signal to promote cell invasion. Inhibition of system x_c_^−^ and AMPARs has been shown to inhibit cell migration, suggesting that autocrine/paracrine signalling is mediated through AMPARs [174]. In addition to conferring a proliferative advantage to the tumour, glutamate excitotoxicity has been proposed to explain the frequent epileptic activity observed in glioma patients (reviewed by Liubinas *et al.* [175]).

## 6. Glutamate Dysregulation in Chronic Pain

Glutamate neurotransmission is fundamental to excitatory signalling in the CNS and thus plays an important role in both normal and pathophysiological nociception. Peripheral sensory information and pain signals are transmitted to the spinal cord via primary afferent neurons, the majority of which are glutamatergic. Upon noxious stimulation, glutamate is released from central terminals in the spinal cord, where it activates AMPARs on secondary neurons. Prolonged activation of nociceptors evokes continuous release of glutamate and subsequently causes long-lasting membrane depolarization. This exaggerated signalling relieves the voltage-dependant magnesium block on NMDARs and consequently allows their activation by glutamate. Additionally, postsynaptically-localised mGluR1, mGluR5, and some presynaptic GluRs localised on central terminals of primary afferents, including kainate receptors (particularly the GluR5 subunit), NMDARs, and some mGluRs, also play a role in nociceptive transmission through feedback mechanisms. Under conditions of increased glutamate release in the spinal cord, including chronic pain, the significance of these receptors increases (Reviewed by Chizh [176]).

Central sensitisation is a phenomenon in which persistent peripheral stimuli or damaged primary afferents trigger a cascade of events that increase the efficacy of glutamatergic neurotransmission in the spinal cord. A key molecular mechanism of central sensitisation is differential sensitisation of AMPARs and NMDARs and enhanced ion channel activity. This central sensitisation is thought to underlie the progression of chronic pain, including effects of allodynia and hyperalgesia [177]. Currently, pharmacological interventions that target GluRs for the treatment of chronic pain are available. NMDAR antagonists, for example, have been used in models of pain induced by inflammation, tissue and nerve injury [178,179], and visceral nociception [180]. Further, data from human patients with neuropathic pain suggest that peripheral NMDARs are involved in nociceptive hypersensitivity [181].

## 7. Oncodynamic Effects on Central Pathologies

### 7.1. Cancer-Induced Depression

As previously mentioned, depression affects approximately 1 in 10 people and contributes a substantial human and financial burden. In cancer patients, the figures are considerably higher. For example, as many as 57% of breast cancer and 93% of high-grade glioma patients report symptoms of depression [182,183]. In addition to the psychosocial impact of receiving a cancer diagnosis, evidence suggests that biological mechanisms contribute to the onset of cancer-induced depression (CID). Clinical studies suggest that depressive symptoms often precede the diagnosis of cancer [184,185,186], with a disproportionally higher number of patients exhibiting depressive symptoms prior to a cancer diagnosis relative to healthy controls [187]. Despite this, pre-clinical efforts to elucidate the biological mechanisms of CID have been sparse, largely owing to the lack of pre-clinical validated models. Recent efforts have addressed this need with the development of a validated mouse model of CID using a murine breast carcinoma cell line [10]. Although mechanisms of CID are in their early stages of investigation, several candidates are proposed based on shared pathophysiological processes between cancer and depression. Notably, cancer and depression are both established to be pro-inflammatory pathologies [188,189,190]. In a mouse model of ovarian cancer, increased levels of interleukin-6 (IL-6) were shown to partially mediate anxiety-like behaviours [191]. Physiological stress is also induced by cancer [192] and strongly implicated in the aetiology of depression, and therefore merits investigation [193]. Glutamatergic dysregulation represents another plausible pathophysiological mechanism for CID. As previously discussed, depression is associated with brain region-specific dysregulation of glutamatergic transmission. Evidence from pharmacological studies using NMDAR antagonists, such as ketamine, to rapidly alleviate depressive behaviours further validate the role of glutamate in the aetiology of depression. Similarly, glioma cells disrupt glutamate transmission in the brain through system x_c_^−^-mediated efflux of glutamate and deficiencies in re-uptake by EAATs. The role of system x_c_^−^ in depression is highlighted by recent evidence showing decreased behavioural despair in a system x_c_^−^ knockout mouse model [194]. It is, therefore, plausible that cancer-mediated glutamate accumulation in the brain interferes with neuroplastic processes in the brain regions that are implicated in depression, such as the PFC and hippocampus. This effect may extend beyond primary brain tumours. As discussed earlier, peripheral cancer cells also release large amounts of glutamate through system x_c_^−^. Under normal physiological conditions, peripheral glutamate is unable to cross the blood-brain barrier (BBB) [195]. However, under the pathological condition of peripheral cancers, the BBB may be weakened, allowing peripheral glutamate to enter the brain. The proinflammatory neuropeptide substance P (SP) is released by cancer cells [196], and has been shown to promote increased permeability of the BBB [197]. Other proinflammatory cytokines that are released by cancers cells, such as IL-17 [198], may play a role in the breaching of the BBB [199]. Therefore, glutamate dysregulation in both central and peripheral cancers may contribute to the aetiological of CID.

### 7.2. Cancer-Induced Bone Pain

Bone is one of the most common sites of metastases, particularly in lung, breast, and prostate cancer [200], occurring in approximately 70% of metastatic breast and prostate cancers [201,202]. The complexity of cancer-induced bone pain (CIBP) is party due to the unique properties of the affected tissue, and is thought to be the result of varied pathological mechanisms. Bone is highly vascularized, and supports a rich neuronal network beneath its surface, among cells that regulate its formation and degradation. Increased osteoclastogenesis results in severe bone destruction and increased exposure of neurovascular beds, leaving them vulnerable to amplified excitatory stimulation.

As previously described, cancer cells that often metastasize to the bone, including breast and prostate carcinomas, release large amounts of glutamate through system x_c_^−^ in the process of taking up cystine for GSH production. This adaptive response to the antioxidant requirements of rapidly-proliferating cancer cells causes disruption in bone metabolic processes (reviewed by Seidlitz *et al.* [203]). Under normal physiological conditions, glutamate has been demonstrated to play an important signalling role in bone formation and resorption [204,205]. Osteoblasts, osteoclasts, and osteocytes have been shown to express iGluRs and mGluRs [204,205,206,207]. In the presence of bone metastases, the balance between bone formation by osteoblasts and degradation by osteoclasts is disturbed [208]. Release of glutamate by cancer cells into the bone microenvironment disrupts this homeostasis, and may contribute to CIBP [209,210,211]. Bone is also known to contain extensive glutamatergic innervation [212]. Consequently, glutamate released by cancer cells may sensitise surrounding nociceptors, directly initiating a pain response in peripheral tissues [203,213]. In addition to directly activating glutamatergic receptors on sensory neurons, the acidity of glutamate has also been shown to stimulate vanilloid receptor 1 (TrpV1), a known nociceptive receptor [214]. In animal models of CIBP, increased expression of spinal cord iGluRs and mGluRs have been reported [215,216]. Pharmacological results from a similar animal model have demonstrated reduced nociceptive behaviors following administration of the system x_c_^−^ inhibitor sulfasalazine [11]. Cumulatively, these results demonstrate that glutamate efflux by bone metastatic cancer cells contributes to CIBP through multiple mechanisms.

## 8. Conclusions

**Figure 1:**
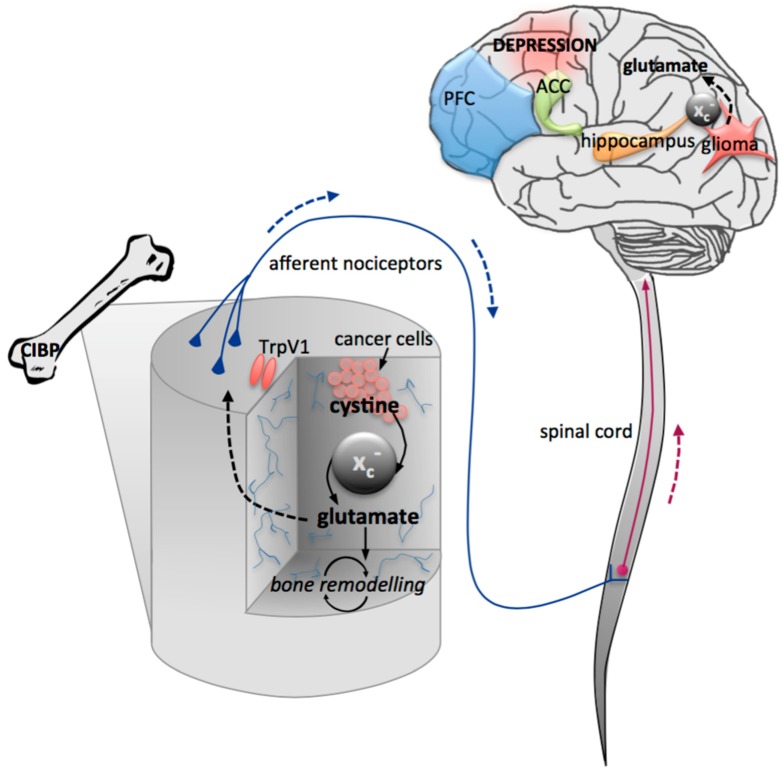
Schematic illustration of the relationship between glutamate dysregulation and central pathologies. Psychiatric disorders, including depression and various anxiety disorders, induce differential glutamate transmission across the brain, particularly in the prefrontal cortex (PFC), anterior cingulate cortex (ACC), and hippocampus. In acute and chronic neurodegenerative conditions, glutamate transport dysregulation is largely attributable to glutamate transporter protein downregulation. The use of pharmacological reagents to strategically manipulate glutamate transmission can elucidate the specific molecular influences of glutamate dysregulation on central pathologies.

Cumulatively, the literature clearly implicates glutamate dysregulation in the progression and maintenance of central pathologies, including psychiatric, neurodevelopmental, and neurodegenerative disorders. An overview of glutamate dysregulation across various pathologies is summarized in Table 1, including the glutamatergic change, associated phenotype, and experimental model employed. Figure 1 illustrates the pathways involved in glutamate dysregulation and indicates the key brain regions that are implicated.

**Table 1:** Glutamate dysregulation in clinical and animal models of psychiatric, neurodevelopmental, and neurodegenerative disorders.

	Pathology	Experimental Model/Intervention	Glutamatergic Change	Phenotype	References	
Psychiatric/Neurodevelopmental	Major depressive disorder	NMDAR antagonist (human and murine)	Activated mTOR pathway, increased BDNF	Reduced depressive symptoms	[41,42,43,44,57]	
		MRS in patients	Decreased glutamate in PFC and ACC		[47,48]	
		Post-mortem patients	Increased AMPAR expression in hippocampus		[49]	
	Bipolar disorder	NMDAR antagonist in patients		Reduced bipolar depression	[67,68]	
		MRS in patients	Decreased glutamate in hippocampus; increased glutamate in ACC, frontal regions, whole brain		[65,69,70]	
		Post-mortem patient hippocampus	Reduced expression of NR1 and NR2A		[72,73]	
	Anxiety	MRS in adolescent GAD patients	Increased Glu/Cr ratio in ACC		[76]	
		NMDAR antagonist in PTSD patients		Reduced anxiety symptoms	[77]	
	Obsessive compulsive disorder	MRS in patients	Decreased glutamate in ACC and OFC		[80,81]	
		Serology in patients	Increased glutamate in CSF		[82,83]	
		NMDAR antagonist in patients		Reduced OCD symptoms	[84]	
		Riluzole in patients	Increased glutamate uptake by astrocytes and inhibited presynaptic glutamate release	Reduced OCD symptoms	[85]	
	Schizophrenia	NMDAR antagonist in healthy subjects		Induced psychotomimetic symptoms	[90,91,92,93]	
		MRS in patients	Increased glutamate in PFC, basal ganglia, hippocampus		[94]	
		Post-mortem patients	Decreased dendritic length, number, spine density, synaptophysin protein expression		[98]	
	Autism spectrum disorder	GRM5 knockout	No expression of mGluR5	Induced ASD behaviours	[104]	
		MRS in patients	Increased glutamate in ACC; decreased glutamate in frontal and occipital lobes		[81]	
	Attention-deficit/hyperactivity disorder	MRS in patients	Increased glutamate in PFC, ACC, striatum		[81]	
		Genome-wide association	GRM7, GRIN2A, GRIN2B, GRID2, EAAT1 polymorphisms	Association with ADHD symptoms	[107]	
		GRM5 knockout/inhibitor	No expression/inhibition of mGluR5	Locomotor hyperactivity, impaired learning	[107]	
Neurodegenerative/Pain	Epilepsy	EAAT2 knockout	Reduced EAAT2 protein	Seizure and death at 6 weeks	[126]
		EAAT3 antisense knockdown	Reduced EAAT3 and GABA	Behavioural abnormalities	[128]
	Alzheimer’s disease	Mutant APP overexpression in transgenic mice	Reduced EAAT1 and EAAT2 protein	Behavioural abnormalities, plaque formation	[109,138]
	Huntington’s disease	Expression of mutant huntington (R6/2)	Reduced EAAT2 protein and mRNA		[111]
	Parkinson’s disease	Disruption of cerebral cortex corpus callosum pathway	Reduced EAAT1 and EAAT2		[112,113]
	Amyotrophic lateral sclerosis	Mutant SOD1 gene	Reduced EAAT2 protein	Paralysis and spinal neuron degeneration	[114,115]
		EAAT2 antisense knockdown	Reduced EAAT2 protein	Paralysis and spinal neuron degeneration	[123]
	Stroke/Ischemia	Hypoxic neonatal pig	Reduced EAAT2 and EAAT3		[120]
		Cortical and hippocampal hypoxia-ischemia	Reduced EAAT1, EAAT2, and EAAT3		[118]
		MCA occlusion	Reduced EAAT1 and EAAT2	Contralateral hemiparesis	[122]
	Chronic pain	NMDAR antagonist		Reduced nociception	[178,179]
	Cancer-induced bone pain	System x_c_^−^ inhibitor	Inhibition of glutamate released by peripheral tumours	Inhibit pain behaviours	[11,171,172]

To understand how glutamate dysregulation contributes to central pathologies, we must first appreciate the influence of other mechanisms involved in disease pathogenesis on glutamate transporter biology. The pathophysiology of glutamate transporter knockout mice suggests that glutamate transporter function is not critical to normal development and does not necessarily signify the development of significant CNS pathology. However, the aberrant functioning of these transporters seemingly contributes to several pathologies in various ways, perhaps by way of introducing a predisposition to insults that would not otherwise produce pathology.

The prevalence of glutamate dysregulation in central pathologies is perhaps not surprising, given its ubiquitous presence in the CNS and its influence as an important neurotransmitter and cell-signalling molecule under normal and pathological conditions. Mounting evidence points to glutamatergic mechanisms as potential targets for pharmacological intervention of central pathologies. The incorporation of pharmacological intervention of glutamate and its transporters into *in vitro* cultures, *in vivo* animal models, and finally, studies of human post-mortem tissues and clinical patients provides the opportunity to elucidate the specific molecular mechanisms of glutamate dysregulation on central pathologies.
